# Supplementary material for: Mechanisms governing the pioneering and redistribution capabilities of the non-classical pioneer PU.1
Source: Nat Commun. 2020 Jan 21;11:402. doi: 10.1038/s41467-019-13960-2 (PMC6972792; doi:10.1038/s41467-019-13960-2)
Supplement: Supplementary file 7 — Source data [file 41467_2019_13960_MOESM7_ESM.zip › Source_Data/FigureS2/FigureS2B_MotifScanOutput/KmeansCluster9/homerResults.html]

/misc/data/analysis/project\_PU1/CTV1/motifs/basic/PU1\_Kmeans14.9/final - Homer de novo Motif Results


# Homer *de novo* Motif Results (/misc/data/analysis/project\_PU1/CTV1/motifs/basic/PU1\_Kmeans14.9/final)

Known Motif Enrichment Results  
Gene Ontology Enrichment Results  
If Homer is having trouble matching a motif to a known motif, try copy/pasting the matrix file into
STAMP  
More information on motif finding results: HOMER
| Description of Results
| Tips
  
Total target sequences = 2276  
Total background sequences = 46142  
\* - possible false positive  

|  |  |  |  |  |  |  |  |  |
| --- | --- | --- | --- | --- | --- | --- | --- | --- |
| Rank | Motif | P-value | log P-pvalue | % of Targets | % of Background | STD(Bg STD) | Best Match/Details | Motif File |
| 1 | A T G C C T G A A T G C C G A T A C G T A G T C A G T C A G C T A G T C G C A T G A T C G C A T | 1e-1190 | -2.741e+03 | 81.20% | 11.31% | 26.2bp (71.1bp) | PU.1(ETS)/ThioMac-PU.1-ChIP-Seq(GSE21512)/Homer(0.965) More Information \| Similar Motifs Found | motif file (matrix) |
| 2 | A C T G C T G A A T G C A C T G C G T A C G T A A T G C | 1e-254 | -5.849e+02 | 50.75% | 18.14% | 46.8bp (72.8bp) | ZNF638(RRM)/Homo\_sapiens-RNCMPT00164-PBM/HughesRNA(0.780) More Information \| Similar Motifs Found | motif file (matrix) |
| 3 | C A G T A T G C G T C A A G T C G C A T C A G T G A T C G T A C A C G T C T A G A G C T | 1e-169 | -3.892e+02 | 14.59% | 1.75% | 46.4bp (67.9bp) | ETS1(ETS)/Jurkat-ETS1-ChIP-Seq(GSE17954)/Homer(0.775) More Information \| Similar Motifs Found | motif file (matrix) |
| 4 | A T G C C G T A A G T C A C G T C A G T T G A C G A T C T A G C G A T C | 1e-117 | -2.703e+02 | 27.15% | 9.59% | 47.4bp (69.4bp) | SpiB(ETS)/OCILY3-SPIB-ChIP-Seq(GSE56857)/Homer(0.756) More Information \| Similar Motifs Found | motif file (matrix) |
| 5 | C T A G G T A C A G T C G C T A A T G C G A T C C G T A A C T G C T G A C A T G A C T G A C T G G T A C T C G A | 1e-91 | -2.112e+02 | 5.32% | 0.27% | 50.7bp (62.5bp) | CTCF/MA0139.1/Jaspar(0.937) More Information \| Similar Motifs Found | motif file (matrix) |
| 6 | T G C A T A G C C G T A C T A G A T C G G T C A C T G A A T C G A C G T C T A G C A T G A C T G C G A T A T C G | 1e-75 | -1.738e+02 | 9.14% | 1.70% | 50.4bp (65.8bp) | ETS:RUNX(ETS,Runt)/Jurkat-RUNX1-ChIP-Seq(GSE17954)/Homer(0.779) More Information \| Similar Motifs Found | motif file (matrix) |
| 7 | C T A G T C A G A T G C C G T A C A T G A C T G C T G A G C T A T C A G A C G T C T A G C T G A | 1e-75 | -1.731e+02 | 9.18% | 1.73% | 55.5bp (66.1bp) | ETS1(ETS)/Jurkat-ETS1-ChIP-Seq(GSE17954)/Homer(0.806) More Information \| Similar Motifs Found | motif file (matrix) |
| 8 | A G T C G A T C T C G A T A G C G C A T G A C T T G A C G A T C G C A T A T G C G T A C G A T C G T C A | 1e-66 | -1.527e+02 | 6.63% | 0.96% | 43.2bp (70.1bp) | ETS:RUNX(ETS,Runt)/Jurkat-RUNX1-ChIP-Seq(GSE17954)/Homer(0.775) More Information \| Similar Motifs Found | motif file (matrix) |
| 9 | T C A G A T G C G A C T A G C T G T A C A G T C C G A T T G A C G A T C T G A C A G T C G A T C | 1e-44 | -1.034e+02 | 9.49% | 2.97% | 47.8bp (67.2bp) | GCR1/Literature(Harbison)/Yeast(0.748) More Information \| Similar Motifs Found | motif file (matrix) |
| 10 | G C A T G C A T C G T A T G C A A T G C T A G C C T G A G T A C G C T A | 1e-40 | -9.396e+01 | 13.09% | 5.38% | 55.5bp (63.4bp) | RUNX1(Runt)/Jurkat-RUNX1-ChIP-Seq(GSE29180)/Homer(0.899) More Information \| Similar Motifs Found | motif file (matrix) |
| 11 | G T C A T G A C C G T A A C T G G C T A A C T G C A T G C G T A C T G A A C T G A C G T A T C G C A T G | 1e-40 | -9.321e+01 | 4.53% | 0.76% | 59.5bp (63.7bp) | PU.1(ETS)/ThioMac-PU.1-ChIP-Seq(GSE21512)/Homer(0.799) More Information \| Similar Motifs Found | motif file (matrix) |
| 12 | T A C G A C T G C A T G T G C A C T G A A T C G C A G T A T C G G A T C C G T A C A T G A C T G C T G A | 1e-33 | -7.771e+01 | 7.51% | 2.43% | 53.7bp (68.7bp) | GFY(?)/Promoter/Homer(0.712) More Information \| Similar Motifs Found | motif file (matrix) |
| 13 | A T G C C T G A T A G C G C A T A C G T A T G C C G A T A T C G G A T C G C A T A G C T G A C T | 1e-33 | -7.697e+01 | 4.17% | 0.80% | 43.0bp (69.6bp) | SpiB(ETS)/OCILY3-SPIB-ChIP-Seq(GSE56857)/Homer(0.836) More Information \| Similar Motifs Found | motif file (matrix) |
| 14 | A T G C G A T C G T C A A T G C G C A T A G C T G A T C A G T C C G A T G A C T G A C T A C G T A G T C | 1e-29 | -6.904e+01 | 2.86% | 0.39% | 54.2bp (67.7bp) | ETS:RUNX(ETS,Runt)/Jurkat-RUNX1-ChIP-Seq(GSE17954)/Homer(0.765) More Information \| Similar Motifs Found | motif file (matrix) |
| 15 | T A C G C T A G A C T G T A C G C G T A C T G A A C T G A C T G T A G C | 1e-27 | -6.368e+01 | 14.89% | 7.80% | 48.9bp (69.9bp) | PCBP2(KH)/Homo\_sapiens-RNCMPT00044-PBM/HughesRNA(0.861) More Information \| Similar Motifs Found | motif file (matrix) |
| 16 | C T A G C T G A C T A G C T A G G T C A C T G A A T C G C T G A C T A G A C T G T C G A | 1e-24 | -5.752e+01 | 9.80% | 4.44% | 42.4bp (77.5bp) | PU.1(ETS)/ThioMac-PU.1-ChIP-Seq(GSE21512)/Homer(0.710) More Information \| Similar Motifs Found | motif file (matrix) |
| 17 | A C T G A T G C C G T A A C T G A C G T A C G T A G T C A G T C A G T C A G C T T G C A T C G A G C T A | 1e-24 | -5.715e+01 | 2.86% | 0.50% | 39.5bp (60.8bp) | ZNF189(Zf)/HEK293-ZNF189.GFP-ChIP-Seq(GSE58341)/Homer(0.720) More Information \| Similar Motifs Found | motif file (matrix) |
| 18 | T A C G G A T C A C T G A T G C G A T C T A G C G A C T T G A C G A C T T C A G A C T G | 1e-24 | -5.687e+01 | 4.66% | 1.33% | 52.7bp (61.3bp) | CTCF/MA0531.1/Jaspar(0.743) More Information \| Similar Motifs Found | motif file (matrix) |
| 19 | C T G A A C G T G T A C G T C A A C T G T A G C G T C A C T G A T C A G A C G T C T A G A C T G | 1e-23 | -5.465e+01 | 5.76% | 1.98% | 58.1bp (64.5bp) | ETS:RUNX(ETS,Runt)/Jurkat-RUNX1-ChIP-Seq(GSE17954)/Homer(0.722) More Information \| Similar Motifs Found | motif file (matrix) |
| 20 | C G A T G T C A G T A C C G A T A C G T T G A C A G T C A G C T C G A T T G A C A G T C | 1e-23 | -5.411e+01 | 7.29% | 2.92% | 47.4bp (97.6bp) | GCR2/MA0305.1/Jaspar(0.673) More Information \| Similar Motifs Found | motif file (matrix) |
| 21 | T A G C G T A C T C A G A G T C G A C T C G T A T C A G | 1e-19 | -4.538e+01 | 39.37% | 30.06% | 56.1bp (63.4bp) | STP3/MA0396.1/Jaspar(0.789) More Information \| Similar Motifs Found | motif file (matrix) |
| 22 | A T G C C T A G A T C G C G T A C G T A G T A C A C G T A C T G | 1e-18 | -4.320e+01 | 8.83% | 4.35% | 46.2bp (64.2bp) | YRM1/MA0438.1/Jaspar(0.786) More Information \| Similar Motifs Found | motif file (matrix) |
| 23 | A C T G A C G T G T C A C G T A A C T G A G C T G C T A C T G A | 1e-17 | -4.102e+01 | 23.55% | 16.25% | 59.4bp (63.5bp) | Scr/dmmpmm(Noyes)/fly(0.749) More Information \| Similar Motifs Found | motif file (matrix) |
| 24 | A C G T A C T G A G C T A T C G A C T G A G T C G T C A | 1e-17 | -4.060e+01 | 28.87% | 20.96% | 56.5bp (62.9bp) | MET32/MA0334.1/Jaspar(0.918) More Information \| Similar Motifs Found | motif file (matrix) |
| 25 | C A G T C T A G G A C T T G C A T G C A C T G A G T A C T C G A | 1e-16 | -3.867e+01 | 34.14% | 25.93% | 59.7bp (64.2bp) | Foxo3(Forkhead)/U2OS-Foxo3-ChIP-Seq(E-MTAB-2701)/Homer(0.960) More Information \| Similar Motifs Found | motif file (matrix) |
| 26 | C T A G T C G A A G T C G A C T C A G T G A T C G A T C A G C T A G T C A G C T A T G C C G T A | 1e-16 | -3.823e+01 | 2.11% | 0.42% | 54.6bp (65.7bp) | PU.1(ETS)/ThioMac-PU.1-ChIP-Seq(GSE21512)/Homer(0.717) More Information \| Similar Motifs Found | motif file (matrix) |
| 27 | G C A T T C A G A T G C A G T C C G T A G A T C A C G T A C G T T A C G A C G T | 1e-16 | -3.800e+01 | 13.18% | 7.88% | 56.2bp (64.1bp) | BHLH112/MA0961.1/Jaspar(0.840) More Information \| Similar Motifs Found | motif file (matrix) |
| 28 | G A C T A G T C G T A C A G T C A C G T T G A C G T A C G T A C G T C A A G T C G T C A | 1e-16 | -3.790e+01 | 4.83% | 1.88% | 53.6bp (66.2bp) | HNRNPH2(RRM)/Homo\_sapiens-RNCMPT00160-PBM/HughesRNA(0.773) More Information \| Similar Motifs Found | motif file (matrix) |
| 29 | A C G T C A T G G T A C C T G A T G C A C G T A A C G T C A T G T G C A | 1e-15 | -3.665e+01 | 16.83% | 10.92% | 56.7bp (64.7bp) | POU2F2/MA0507.1/Jaspar(0.838) More Information \| Similar Motifs Found | motif file (matrix) |
| 30 | C T G A T C A G T C G A T C G A C G T A C T A G T A C G G T A C C T G A T C A G C A G T T A C G C G T A C T A G | 1e-15 | -3.539e+01 | 0.83% | 0.04% | 52.3bp (94.3bp) | Tb\_0220(RRM)/Trypanosoma\_brucei-RNCMPT00220-PBM/HughesRNA(0.769) More Information \| Similar Motifs Found | motif file (matrix) |
| 31 | C G T A A G C T A G C T C G T A A T C G G C T A A C G T T C A G C A T G A G T C | 1e-15 | -3.467e+01 | 3.34% | 1.09% | 61.6bp (66.2bp) | NeuroD1(bHLH)/Islet-NeuroD1-ChIP-Seq(GSE30298)/Homer(0.665) More Information \| Similar Motifs Found | motif file (matrix) |
| 32 | C G A T A G C T C G T A C G A T G A T C G T C A A C T G T G A C G T A C | 1e-14 | -3.349e+01 | 18.37% | 12.46% | 58.6bp (63.1bp) | Unknown5/Drosophila-Promoters/Homer(0.853) More Information \| Similar Motifs Found | motif file (matrix) |
| 33 | C A G T G A T C C G T A C A G T A T C G A C G T A C T G T C A G A C G T A C G T A C G T C G A T C T G A C G T A | 1e-13 | -3.081e+01 | 0.70% | 0.03% | 49.6bp (48.5bp) | RUNX1(Runt)/Jurkat-RUNX1-ChIP-Seq(GSE29180)/Homer(0.685) More Information \| Similar Motifs Found | motif file (matrix) |
| 34 | C A G T C T A G C T G A C T G A C G T A A C T G T C G A A C T G G T A C G T A C G C T A A C T G A G C T C A G T | 1e-12 | -2.827e+01 | 0.70% | 0.04% | 60.8bp (46.0bp) | brk/dmmpmm(Down)/fly(0.610) More Information \| Similar Motifs Found | motif file (matrix) |
| 35 | A T C G C T G A A G C T C G T A C A G T A G T C G A C T A C T G C T A G C T A G A T C G C G T A C G T A C T G A | 1e-12 | -2.815e+01 | 0.79% | 0.06% | 54.6bp (69.4bp) | MIG1(MacIsaac)/Yeast(0.677) More Information \| Similar Motifs Found | motif file (matrix) |
| 36 | A T G C G T C A C T G A C G T A A C G T A C T G G T A C G A T C G T C A A G T C C G T A A C T G A T C G A G T C | 1e-12 | -2.802e+01 | 0.62% | 0.03% | 57.3bp (60.0bp) | CST6(MacIsaac)/Yeast(0.667) More Information \| Similar Motifs Found | motif file (matrix) |
